# Effect of Au Nanoparticles and Scattering Layer in Dye-Sensitized Solar Cells Based on Freestanding TiO_2_ Nanotube Arrays

**DOI:** 10.3390/nano11020328

**Published:** 2021-01-27

**Authors:** Kang-Hun Lee, Seung-Hee Han, Ana Chuquer, Hwa-Young Yang, Jaehi Kim, Xuan-Hung Pham, Won-Ju Yun, Bong-Hyun Jun, Won-Yeop Rho

**Affiliations:** 1 School of International Engineering and Science, Jeonbuk National University, 567, Baekje-daero, Deokjin-gu, Jeonju-si, Jeollabuk-do 54896, Korea; dlrkdgns343@naver.com (K.-H.L.); rinmin0616@naver.com (S.-H.H.); 2 School of Bioenvironmental Chemistry, Jeonbuk National University, 567, Baekje-daero, Deokjin-gu, Jeonju-si, Jeollabuk-do 54896, Korea; chuquerana@gmail.com; 3 School of Energy and Chemical Engineering, Ulsan National Institute of Science and Technology (UNIST), 50 UNIST-gil, Eonyang-eup, Ulju-gun, Ulsan 44919, Korea; hyang@unist.ac.kr; 4 Department of Bioscience and Biotechnology, Konkuk University, 120, Neungdong-ro, Gwangjin-gu, Seoul 05029, Korea; susia45@gmail.com (J.K.); phamricky@gmail.com (X.-H.P.); 5 Department of Physics, Jeonbuk National University, 567, Baekje-daero, Deokjin-gu, Jeonju-si, Jeollabuk-do 54896, Korea; enneades@hanmail.net

**Keywords:** Au nanoparticles, TiO_2_ nanotube arrays, electron generation, plasmonic effect, scattering layer, light harvesting

## Abstract

The development of high efficiency dye-sensitized solar cells (DSSCs) has received tremendous attention. Many researchers have introduced new materials for use in DSSCs to achieve high efficiency. In this study, the change in power conversion efficiency (PCE) of DSSCs was investigated by introducing two types of materials—Au nanoparticles (Au NPs) and a scattering layer. A DSSC fabricated without neither Au NPs nor a scattering layer achieved a PCE of 5.85%. The PCE of a DSSC based on freestanding TiO_2_ nanotube arrays (f-TNTAs) with Au NPs was 6.50% due to better electron generation because the plasmonic absorption band of Au NPs is 530 nm, which matches the dye absorbance. Thus, more electrons were generated at 530 nm, which affected the PCE of the DSSC. The PCE of DSSCs based on f-TNTAs with a scattering layer was 6.61% due to better light harvesting by scattering. The scattering layer reflects all wavelengths of light that improve the light harvesting in the active layer in DSSCs. Finally, the PCE of DSSCs based on the f-TNTAs with Au NPs and a scattering layer was 7.12% due to the synergy of better electron generation and light harvesting by plasmonics and scattering. The application of Au NPs and a scattering layer is a promising research area for DSSCs as they can increase the electron generation and light harvesting ability.

## 1. Introduction

Solar cells are energy generating devices that are of interest because of their safe, clean, and eco-friendly features. Dye-sensitized solar cells (DSSCs) are a particular kind of solar cell that has been researched since they were first studied by O’Regan and Grätzel [1]. After their discovery, DSSCs attracted a lot of attention due to their efficiency and ease of implementation. Compared with previous generations, DSSCs have improved and have many beneficial properties such as low cost, low toxicity, a semi-flexible structure, and light weight [2,3,4]. They are made using a simple process and work perfectly in low light intensity environments. For all these reasons, DSSCs are promising next generation solar cells.

DSSCs commonly consist of a semiconductor made of TiO_2_, an electrolyte, dye molecules, and a counter electrode. There are two kinds of TiO_2_ electrodes based on structure. The first one contains a mesoporous TiO_2_ film that consists of nanosized TiO_2_ [5,6]. In this structure, the surface areas of mesoporous TiO_2_ films are high. Therefore, a lot of dye can be absorbed on the film, but these films inevitably degrade the performance of DSSCs due to defects in structure, numerous trapping sites and grain boundaries, which impede electron transportation [7,8,9,10]. Second, by using an anodization electrochemical method, TiO_2_ nanotube arrays (TNTAs) possess a vertical structure which is highly ordered. The surface area of TNTAs is relatively small compared with mesoporous TiO_2_ films, so less dye can be attached to TNTAs. However, the loss of electrons and charge recombination are smaller than mesoporous TiO_2_ films as the electrons move to counter electrodes due to the vertical structure of TNTAs [11,12,13,14,15,16,17,18]. For these reasons, the application of TNTAs can improve the power conversion efficiency (PCE) of DSSCs compared to the application of mesoporous TiO_2_ NP films.

Plasmonic nanoparticles (NPs) are strong absorbers and can scatter light due to the surface plasmon resonance (SPR) phenomenon [19,20]. SPR is defined as the collective oscillation of conduction electrons at the surface of a metallic nanostructure. Plasmonic NPs are of interest because of unique optical properties. For example, Ag NPs have high electrical conductivity and good stability. Cu NPs also have high conductivity but are unstable. Among other metallic NPs, Au NPs are more chemically stable and have a lower energy band [21,22,23,24,25,26]. The scattering layer in DSSCs collects light and improves light harvesting due to the reflection of the light. ZrO_2_ and SiO_2_, which have a good reflectance behavior, can be used as the scattering layer, but TiO_2_ has special features. Hence, a TiO_2_ light scattering layer can enhance light absorption, and thus support adequate light trapping—reducing charge recombination [27,28,29,30,31,32,33].

In this study, DSSCs were fabricated with freestanding TNTAs (f-TNTAs) with Au NPs and a scattering layer instead of mesoporous TiO_2_ films for better performance of DSSCs. Au NPs were introduced into the f-TNTAs to increase electron generation by plasmonics, and a TiO_2_ scattering layer was also introduced to enhance light harvesting.

## 2. Materials and Methods

### 2.1. Preparation of f-TNTAs

A Ti plate (99.7%, 2.5 cm × 4.0 cm × 100 μm, Alfa) was sonicated with deionized water, ethanol, and acetone for 30 min to remove surface contamination. The electrolyte for anodization consisted of H_2_O (2 vol. %) and NH_4_F (0.8 wt. %) in ethylene glycol. The 1st Ti anodization was performed in the electrolyte at 25 °C with an applied 60 V DC for 2 h. The 1st TNTAs on the Ti plate was sintered at 500 °C for 1 h under the ambient condition. After the sintering process, the 2nd Ti anodization was applied at the same temperature as the 1st anodization under the 30 V DC for 10 min. This procedure results in 2nd generation TNTAs between the 1st TNTAs and Ti plate. To obtain the 1st TNTAs, the 2nd TNTAs were removed by dipping the sample in a 10% of H_2_O_2_ solution for 1 h.

### 2.2. Preparation of Au NPs into the f-TNTAs

The TiO_2_ compact layer was coated on fluorine-doped tin oxide (FTO) glass with a precursor solution which consists of 5 wt. % titanium di-isopropoxide bis(acetylacetonatei) (75 wt. % in isopropanol, Aldrich, St. Louis, MI, USA) in butanol. The mesoporous TiO_2_ film was doctor-bladed using paste (Ti-Nanoxide T/SP, Solaronix, Aubonne, Switzerland), and then f-TNTAs were transferred onto the TiO_2_ film. Subsequently, the film was sintered at 500 °C for 1 h to enhance the adhesion between f-TNTAs and a mesoporous TiO_2_ film. In the channel of f-TNTAs, Au NPs were loaded by an electrochemical deposition method in 10 mM of HAuCl_4_ (99.99%, Aldrich, St. Louis, MI, USA) was mixed with a 50/50 (*v*/*v*) solution of ethanol and H_2_O with an applied voltage of 5 V DC.

### 2.3. Fabrication of DSSC f-TNTAs with a Scattering Layer

The TiO_2_ paste (Ti-Nanoxide R/SP, Solaronix, Aubonne, Switzerland) was doctor-bladed on a substrate that contained Au NP loaded f-TNTAs, and it was subsequently sintered at 500 °C for 1 h under ambient conditions. The Au NP decorated f-TNTAs with a scattering layer were treated with TiCl_4_ by soaking the substrate in a 0.01 M TiCl_4_ solution at 50 °C for 30 min, and they were sintered at 500 °C for 1 h.

### 2.4. Fabrication of DSSCs with Au NPs Decorated into f-TNTAs

The substrate that consists of a scattering layer and Au NP-loaded f-TNTAs was soaked in a ruthenium dye (Bu_4_N)_2_Ru(dcbpyH)_2_(NCS)_2_ (N719, Solaronix, Aubonne, Switzerland) solution at 50 °C for 8 h. Eventually, the substrate was fully coated with N719 and was used as the working electrodes of DSSCs. The platinum (Pt) layer was prepared by dropping 0.5 mM of a H_2_PtCl_6_·6H_2_O (Aldrich, St. Louis, MI, USA) solution on the FTO glass, followed by heating at 300 °C for 30 min as a counter electrode. Each of the counter and working electrode were assembled into a sandwich-type cell and were sealed using a hot-melt film (Surlyn-1702, Dupont, Mississauga, ON, Canada) on a hotplate. The space between the two electrodes was filled with an electrolyte containing 0.7 M 1-butyl-3-methyl-imidazolium iodide (BMII, 99%, Aldrich, St. Louis, MI, USA), 0.03 M iodine (99%, Aldrich, St. Louis, MI, USA), 0.1 M guanidium thiocyanate (GSCN, 99%, Aldrich, St. Louis, MI, USA), and 0.5 M 4-tertbutylpyridine (TBP, 96%, Aldrich, St. Louis, MI, USA) in an 85/15 (*v*/*v*) solution of acetonitrile and valeronitrile.

### 2.5. Characterization

Under AM 1.5 illuminated condition (150 W, xenon solar simulator, 91160 A), the I–V characteristic measurements of DSSCs were obtained by Keithley 2400. The morphologies and sizes of TNTAs were assessed using a scanning electron microscope (FE-SEM, SU-70, Hitachi, Tokyo, Japan). Decorated Au NPs in the f-TNTAs were confirmed by transmission electron microscopy (Cs-FE-TEM, JEM-ARM-200F, JEOL Inc. Peabody, MA, USA) and X-ray diffractometry (XRD, Max-2500, Rigaku, Tokyo, Japan). The incident photon-to-current conversion efficiency (IPCE) were measured in monochromatic (MonoRa150i, Dongwoo Optron, Gwangju, South Korea) light using a 150 W xenon lamp (LS150, Abet Technologies, Milford, CT, USA) with source measure units (CompactStat, Ivium, Eindhoven, The Netherlands). Electrochemical impedance spectroscopy (EIS) was carried out using a potentiostat and frequency response analyzer (SI 1287, SI 1260, Solartron Analytical, Southern Pines, NC, USA) between 10^−2^ and 10^6^ Hz and an AC amplitude of 10 mV at the *V_oc_* of the DSSC under AM 1.5 light illumination. The impedance spectra were analyzed using a software (Z-View, Scribner Associates, Southern Pines, NC, USA) for appropriate fitting of the EIS data to an equivalent circuit.

## 3. Results and Discussion

**Figure 1:**
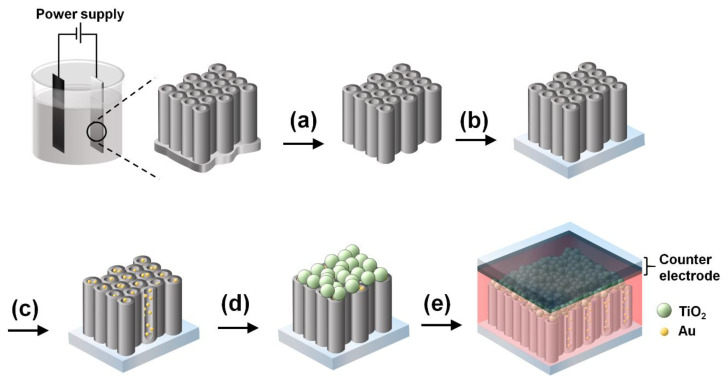
Overall scheme for fabrication of dye-sensitized solar cells (DSSCs) based on the freestanding TiO_2_ nanotube arrays (f-TNTAs) with Au nanoparticles (Au NPs) and a scattering layer. (a) Preparation of freestanding TiO_2_ nanotube arrays (f-TNTAs), (b) transfer of f-TNTAs on FTO glass, (c) electrodeposition of Au NPs into the f-TNTAs, (d) introduction of the scattering layer, (e) fabrication of DSSCs.

Figure 1 explains the preparation of DSSCs with Au NP-decorated f-TNTAs and a scattering layer. The f-TNTAs were synthesized by two steps of Ti plate anodization—1st anodization and 2nd anodization. After the 1st anodization, part of the Ti plate becomes 1st generation TNTAs, and those TNTAs are crystalline [34,35,36,37,38]. The 1st TNTAs were stable in basic or acidic solutions, so a 2nd anodization was needed to obtain f-TNTAs. The 2nd TNTAs had an amorphous phase that was weak under acidic or basic conditions. Thus, the 2nd TNTAs were easily removed by H_2_O_2_ solution, which detached the 1st TNTAs from the Ti plate (referred to as f-TNTAs as shown in Figure 1 (a)). Prepared f-TNTAs were transferred to the FTO glass with TiO_2_ paste and were attached by an annealing at 500 °C as shown in Figure 1 (b). Au precursor was introduced into the inner part of the f-TNTAs by immersing f-TNTAs in an Au solution and applying a constant voltage of 5 V DC as shown in Figure 1 (c) [39,40,41]. Using a doctor blade, the TiO_2_ scattering layer was coated on the f-TNTAs as shown in Figure 1 (d). The DSSCs were fabricated with Au NP-embedded f-TNTAs and a TiO_2_ scattering layer. The FTO glass with Au NP-decorated f-TNTAs and a TiO_2_ scattering layer is called the “working electrode” and the FTO coated with Pt is called the “counter electrode.” The electrolyte was filled into the space between the working and counter electrode, and then DSSCs were fabricated as shown in Figure 1 (e).

**Figure 2:**
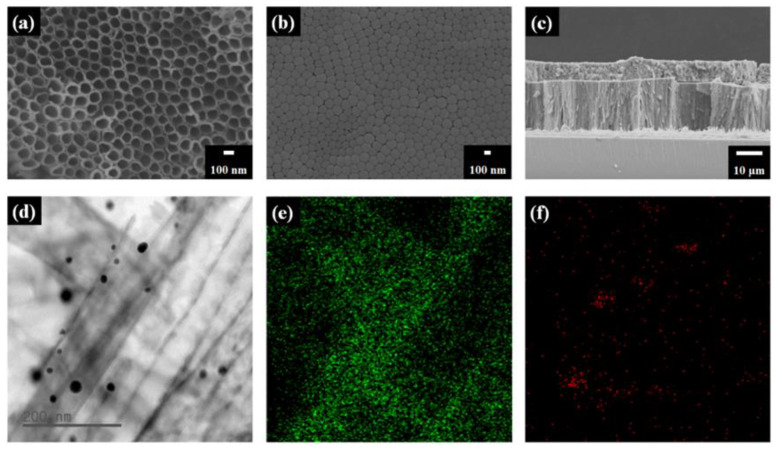
Field-emission scanning electron microscopy (FE-SEM) images of (**a**) top view of f-TNTAs, (**b**) bottom view of f-TNTAs, (**c**) side view of scattering layer and f-TNTAs by FE-SEM, (**d**) transmission electron microscopy (TEM) image of f-TNTAs with Au NPs, (**e**) element mapping of Ti (green) from TiO_2_, and (**f**) element mapping of Au (red) from Au NPs.

The f-TNTAs were characterized using field-emission scanning electron microscopy (FE-SEM) as shown in Figure 2a. The pore sizes of f-TNTAs after anodization were approximately 100 nm. Figure 2b shows the bottom layer of f-TNTAs after separation from the Ti plate, and the pore diameters were also approximately 100 nm. Figure 2c shows the side view of f-TNTAs and a scattering layer. The lengths of the f-TNTAs were approximately 18 μm, and the height of the scattering layer was about 10 μm. The Au NPs were well decorated into the f-TNTAs and were characterized using Cs-corrected-field emission transmission electron microscopy (Cs-FE-TEM) as shown in Figure 2d. The black lines are the walls of f-TNTAs, and the small black dots are Au NPs. Figure 2e,f are the element mapping images of Ti and Au NPs. The green elements represent Ti from f-TNTAs, and the red elements are Au from Au NPs. The green is observed at the wall positions of Ti from f-TNTAs, and the red dots are the same as the position of Au NPs.

**Figure 3:**
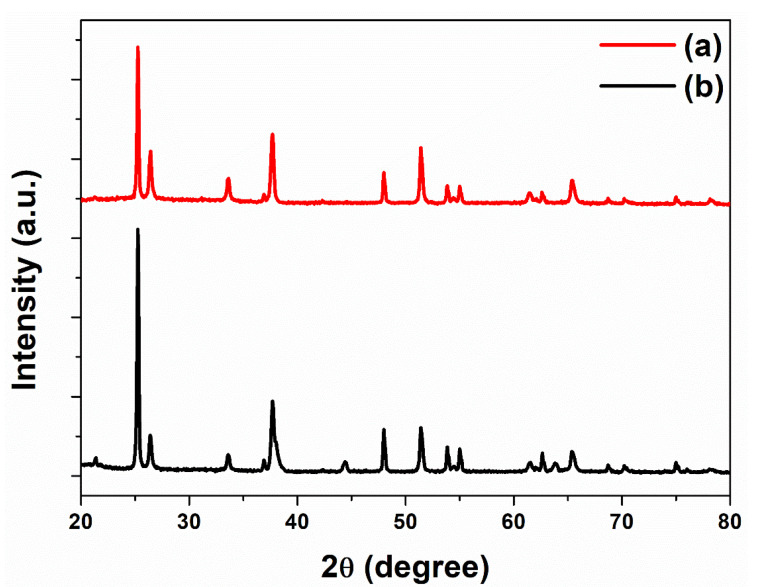
X-ray diffraction (XRD) data for: (a) f-TNTAs and (b) f-TNTAs with Au NPs.

The crystal phase structures of f-TNTAs and Au NPs were analyzed by X-ray diffraction (XRD). The XRD patterns of each f-TNTAs and Au NPs are shown in Figure 3. The red line represents the XRD pattern of the freestanding TNTA phase at 2 θ = 25.5°, 38.2°, 48.0°, 54.1°, 55.2°, 62.8°, 70.3°, and 75.1° corresponding to the (101), (004), (200), (105), (211), (204), (220), and (215) planes of the anatase f-TNTAs. The black line (in which the XRD pattern of Au NPs are decorated into the f-TNTAs) shows three different peaks compared with the red line. The wide range peak at a 2 θ of 38.3° (covered by peaks of anatase TiO_2_ at a 2 θ of 38.2°) and two narrow range peaks at 2 θ values of 44.3° and 64.5° correspond to (111), (200), and (200) planes of Au NPs. These XRD results verified that Au NPs were well-decorated into the f-TNTAs by the electrochemical deposition method.

**Figure 4:**
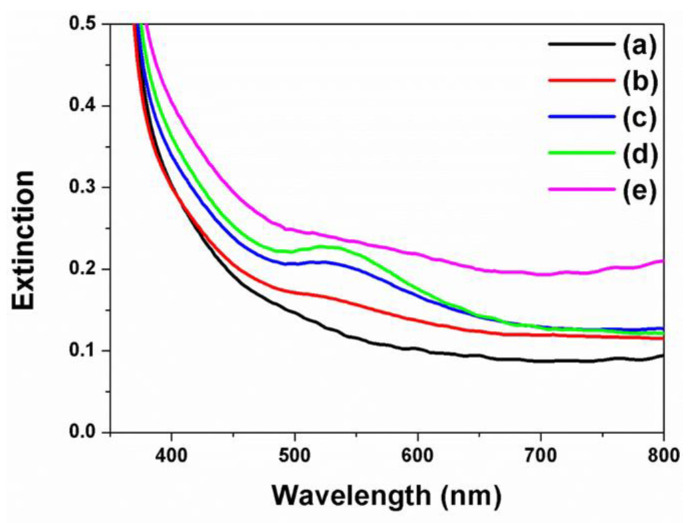
UV-vis absorption spectra of f-TNTAs with Au NPs decorated by electrochemical deposition method for (a) 0 s, (b) 10 s, (c) 20 s, (d) 30 s, and (e) 40 s.

Figure 4 shows the UV-vis spectra results of f-TNTAs with Au NPs deposited by the electrochemical deposition method for 0, 10, 20, 30, and 40 s. Figure 4 (a) represents the UV-vis results of f-TNTAs without Au NPs, and the graph flows smoothly through 400–800 nm. When using f-TNTAs with Au NP sizes of 5–80 nm, the extinction peaks were excited from 530–700 nm [42,43]. Those extinction peaks show that the Au NPs were well matched to N719 dye, and the absorbance range of DSSCs was from 340 to 540 nm. When the Au NPs were decorated into f-TNTAs via an electrochemical deposition method for 10 s, a slight peak was observed at 530 nm, as shown in Figure 4 (b). The peaks at 530 nm became stronger by increasing the electrochemical deposition time from 20 to 30 s. This confirmed that the amount of Au NPs could be controlled by changing the reaction time with increasing electrochemical deposition time. The strongest signal was shown on Figure 4 (d), and this was for DSSCs when Au NPs were decorated into f-TNTAs for 30 s. In the case of 40 s, there was no significant peak at 530 nm in Figure 4 (e). The large amount of Au NPs at longer electrochemical deposition times caused aggregation of Au NPs, resulting in extinction that did not correspond to the absorbance of the N719 dye.

**Figure 5:**
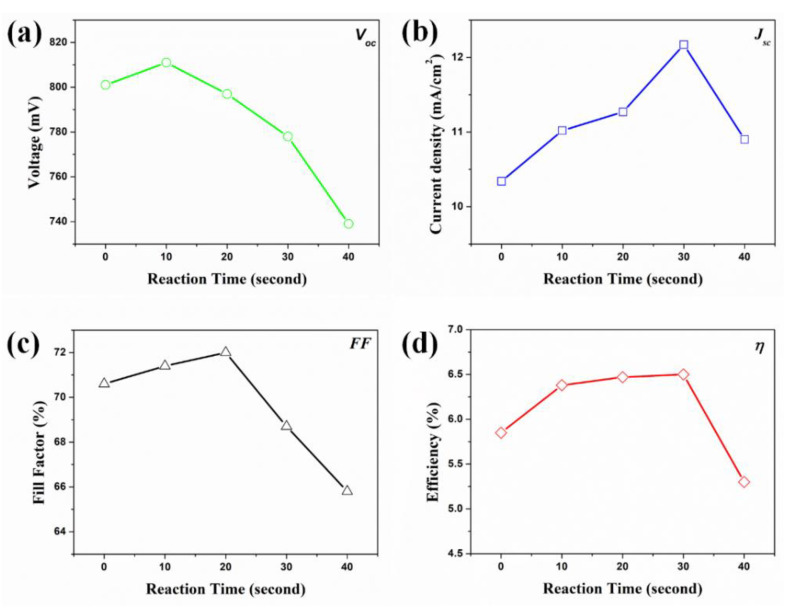
Photovoltaic properties of DSSCs based on the f-TNTAs with Au NPs: (**a**) voltage, (**b**) current density, (**c**) fill factor, and (**d**) efficiency.

The photovoltaic properties of DSSCs based on f-TNTAs with Au NPs were measured under an AM 1.5, one-sun condition, and the results are shown in Figure 5 as open-circuit voltage (*V_oc_*) (a), short-circuit current density (*J_sc_*) (b), fill factor (*FF*) (c), and power conversion efficiency (PCE, *η*) (d). These factors are summarized in Table 1. The amount of Au NPs depended on the reaction time. The plasmonics will distinguish the “charging effect” and “plasmonic effect” because the Au NPs have metal properties [21]. Electrons were trapped by Au NPs when relatively few Au NPs were decorated into the active layer of DSSCs. Thus, the electron density on the active layer increased, which affected the Fermi level. Consequently, the *V_oc_* and *FF* increased via the “charging effect.” When an optimized amount of Au NPs was decorated into the active layer of DSSCs, many electrons were generated by Au NPs due to the surface plasmon resonance (SPR) phenomenon, which affected the *J_sc_* and *FF* via the “plasmonic effect.” As shown in Figure 5a, as the reaction time increased from 0 s to 40 s, the DSSC showed the highest *V_oc_* at 10 s due to the charging effect. As the reaction time increased, the *V_oc_* of the DSSC gradually decreased because of the recombination. As shown in Figure 5b, the *J_sc_* gradually enhanced with increasing reaction time until 30 s, and it reached the highest *J_sc_* at 30 s due to the plasmonic effect. In Figure 5c, DSSC had the highest *FF* at 20 s due to the synergy of the charging effect and plasmonic effect. The charging effect is related to *V_oc_*, the plasmonic effect is related to *J_sc_*, and the electron density is related to *FF*. Thus, the electron density is determined by the charging effect and plasmonic effect. As shown in Figure 5d, the DSSC had a better PCE at 30 s in spite of a decrease in the *V_oc_*. In a DSSC with a 30 s reaction time, the electrons were recombined via Au NPs using the charging effect, but more electrons were generated by Au NPs via the plasmonic effect. Therefore, the net electron density on the active layer increased. Finally, the total PCE of the DSSC was higher than any other DSSCs at 30 s. As shown in Figure 5a–d, the DSSCs had low values of *V_oc_*, *J_sc_*, *FF,* and PCE at 40 s. This is because the Au NPs were aggregated, making their main role active layer recombination. So, the electron density on the active layer decreased, resulting in the low *V_oc_*, *J_sc_*, *FF*, and PCE.

**Figure 6:**
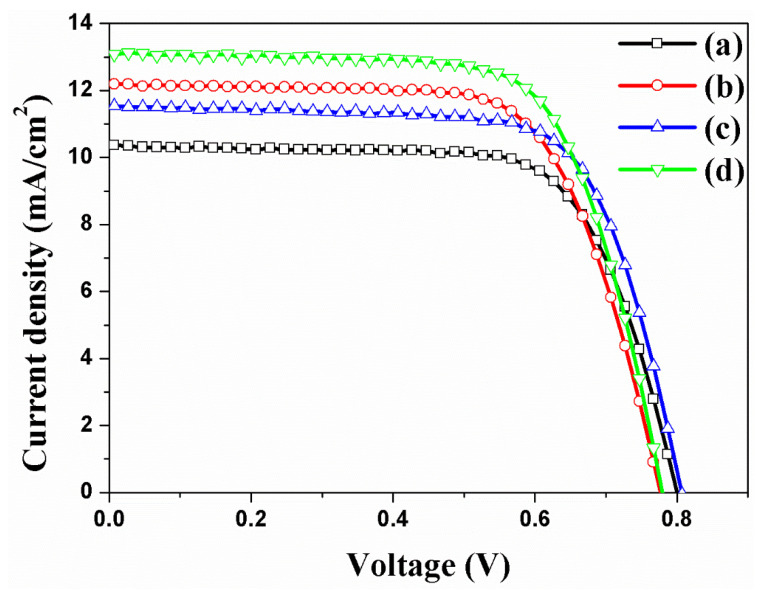
I–V curves of DSSCs based on the f-TNTAs: (a) without both Au NPs and a scattering layer, (b) with Au NPs, (c) with a scattering layer, and (d) with Au NPs and a scattering layer.

PCEs of four kinds of DSSCs were characterized under one sun conditions: DSSCs based on the f-TNTAs (a) without Au NPs and without a scattering layer, (b) with Au NPs, (c) with a scattering layer, and (d) with Au NPs and a scattering layer. Summarized results of the short-circuit density (*J_sc_*), open-circuit voltage (*V_oc_*), fill factor (*FF*), and PCE (*η*) of each DSSC are shown in Figure 6 and Table 2. First, the PCE of DSSCs based on f-TNTAs without Au NPs and without a scattering layer was 5.85%. Second, the PCE of DSSCs based on f-TNTAs with Au NPs was 6.50%, an enhancement of 11.11% compared to the PCE of DSSCs based on the f-TNTAs without Au NPs and a scattering layer. The *J_sc_* of DSSCs based on f-TNTAs with Au NPs increased from 10.34 to 12.17 mA/cm^2^ as compared to the DSSCs based on f-TNTAs without Au NPs and a scattering layer because of the number of electrons generated by plasmonics. However, the *V_oc_* and *FF* decreased by Au NPs due to the recombination. Third, the PCE increased to 6.61%, an enhancement of 12.99%, by introducing a scattering layer on the DSSCs. Introducing a scattering layer on the DSSCs increased light harvesting ability due to the scattering layer, which helped collect the light efficiently. Additionally, the scattering layer provided an additional high surface area that could provide high dye absorption. For this reason, DSSCs based on the scattering layer have enhanced current density and PCE compared to DSSCs based on the TiO_2_ NPs layer as shown in Figure S1 and Table S1. Finally, the PCE of DSSCs based on f-TNTAs with Au NPs and a scattering layer increased to 7.12%, an enhancement 21.70% compared to the PCE of DSSCs based on the f-TNTAs without Au NPs and a scattering layer. The Au NPs increased the electron generation by plasmonic effects, and the TiO_2_ scattering layer increased the light harvesting ability by diffusing the light.

**Figure 7:**
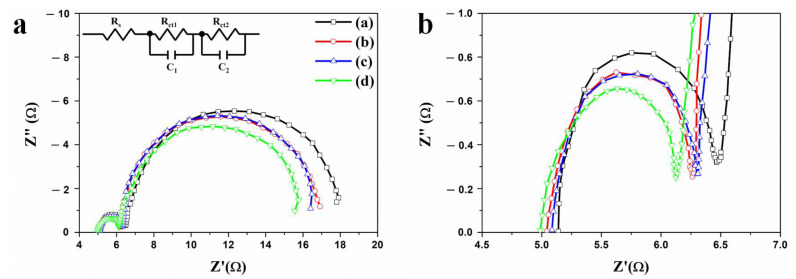
Impedance of DSSC based on the f-TNTAs is (**a**), and the expanded small semicircles of the impedance spectrum from (**a**) is shown in (**b**): (a) f-TNTAs without both Au NPs and a scattering layer, (b) f-TNTAs with Au NPs, (c) f-TNTAs with a scattering layer, and (d) f-TNTAs with Au NPs and a scattering layer.

Electrochemical impedance spectroscopy (EIS) was used to measure the resistances to confirm the effect of Au NPs and a scattering layer in DSSCs. The impedance spectrum of a DSSC based on f-TNTAs with Au NPs and a scattering layer is shown in Figure 7. The fit parameters of the impedance properties are summarized in Table 3, and they contain *R_s_*, *R_1_*, and *R_2_*. Each spectrum contains two differently sized semicircles. *R_s_* is the series resistance of DSSCs whose starting point is semicircles on the x-axis. As the current density (*J_sc_*) of DSSCs increased by improving charge transport, the series resistance *R_s_* decreased. The *J_sc_* values of DSSCs based on f-TNTAs: (a) without Au NPs and without a scattering layer, (b) with Au NPs, (c) with a scattering layer, and (d) with Au NPs and a scattering layer were 10.34, 12.17, 11.56, and 13.10 mA/cm^2^, corresponding to *R_s_* values of 5.099, 5.039, 5.059, and 4.942 Ω, respectively. The increased charge transport increases *J_sc_*, which also decreases *R_s_* in DSSCs. *R_1_* is the sum of the small semicircle at high frequency, which represents the interfacial resistance of FTO/f-TNTAs and Pt/electrodes in DSSCs as shown in Figure 7 (b). The interfacial resistance (*R_1_*) values of FTO/f-TNTAs and Pt/electrodes in DSSCs: (a) without Au NPs and without a scattering layer, (b) with Au NPs, (c) with a scattering layer, and (d) with Au NPs and a scattering layer were 1.257, 1.088, 1.098, and 1.071 Ω. In DSSCs with Au NPs and/or a scattering layer, more electrons were generated by plasmonics and light harvesting, which decreased the interfacial resistance of FTO/f-TNTAs. *R_2_* is the sum of large semicircles at low frequency, which represents the interfacial resistance of f-TNTAs/electrolyte and electron transport or transfer resistance of f-TNTAs. The *R_2_* values of DSSCs: (a) without Au NPs and without a scattering layer, (b) with Au NPs, (c) with a scattering layer, and (d) with Au NPs and a scattering layer were 12.77, 11.68, 11.75, and 10.98 Ω, respectively. More electrons were generated by plasmonics in DSSCs with Au NPs or/and a scattering layer, and light harvesting that improved the electron transport or transfer on f-TNTAs in DSSCs. Electrochemical impedance data showed that the electron generation and light harvesting ability were improved by the introduction of Au NPs and a scattering layer in DSSCs.

**Figure 8:**
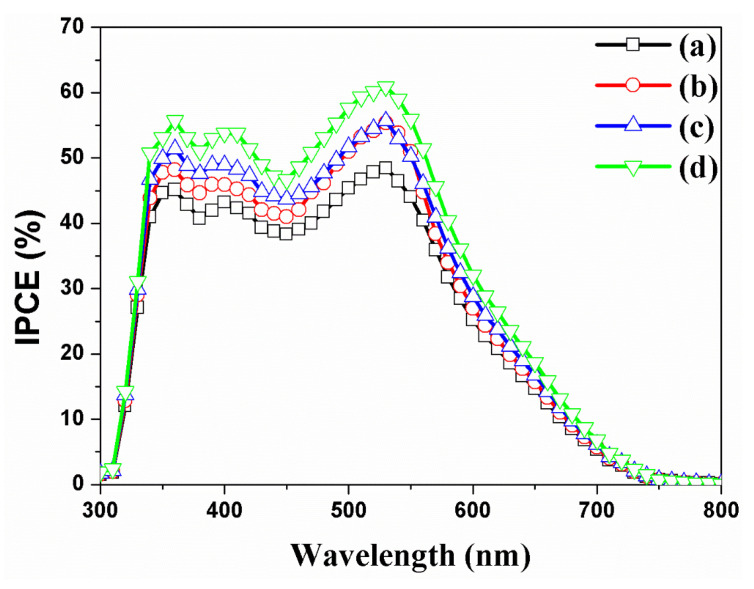
Incident photon-to-current conversion efficiency (IPCE) of DSSC based on the f-TNTAs (a) without both Au NPs and a scattering layer, (b) with Au NPs, (c) with a scattering layer, and (d) with Au NPs and a scattering layer.

Incident photon to electron conversion efficiency (IPCE) data were obtained for DSSCs with/without Au NPs and TiO_2_ scattering layers. DSSCs without Au NPs and a scattering layer showed absorption bands of N719 dye at 393 and 533 nm as shown in Figure 8 (a). After the Au NPs were decorated into the f-TNTAs in a DSSC as shown in Figure 8 (b), the IPCE data showed an increase at 530 nm compared to DSSCs without Au NPs and a scattering layer. This is because the current density of the DSSCs with Au NPs was increased by the plasmonics at 530 nm. The UV-vis spectra confirmed that introducing Au NPs resulted in some special extinction at 530 nm. After the scattering layer was introduced on the f-TNTAs in the DSSC, as shown in Figure 8 (c), the IPCE data showed a uniform increase from 390 to 530 nm. This is different from the case in which Au NPs were decorated into the f-TNTAs shown in Figure 8 (b) because the current density of the DSSCs with a scattering layer increased due to light harvesting. Finally, after both Au NPs and a scattering layers were introduced in DSSCs, Figure 8 (d), the IPCE data showed much better efficiency than any other results due to the plasmonics and light harvesting.

## 4. Conclusions

Well-dispersed Au NPs with a plasmonic absorption band of 530 nm decorated in f-TNTAs by an electrochemical method were used instead of mesoporous TiO_2_ for better electron generation in DSSCs. The amount of Au NPs affected the *V_oc_, FF,* and *J_sc_*. When Au NPs were decorated into the f-TNTAs for 10 s, the *V_oc_* of DSSCs was higher than any other DSSCs due to charging effects. However, when Au NPs were decorated into the f-TNTAs for 30 s, the *J_sc_* of the DSSC was higher than any other DSSCs due to the plasmonic effect. Consequently, the PCE of a DSSC with Au NP-decorated f-TNTAs increased from 5.85% to 6.50%, an enhancement of 11.11% compared to the PCE of a DSSC without Au NPs decorated in f-TNTAs due to charging and plasmonic effects. The scattering layer reduced the amount of light loss by changing light to irregular angles, and it enhanced the light harvesting ability of DSSCs. The PCE of DSSCs with a scattering layer increased from 5.85% to 6.61%, an enhancement of 12.99% compared to the PCE of DSSCs without a scattering layer. Finally, both Au NP-decorated f-TNTAs and the scattering layer were introduced into DSSCs, and the PCE reached 7.12%, an enhancement of 21.70%. These methods could be used for other kinds of solar cells such as silicon solar cells, perovskite solar cells, and organic/inorganic solar cells in future investigations.

## Figures and Tables

**Table 1:** Photovoltaic properties of DSSCs based on f-TNTAs with Au NPs.

DSSCs Based on the f-TNTAs with Au NPs		*J_sc_* (mA/cm^2^)	*V_oc_* (mV)	*FF* (%)	*η* (%)	Dye Loading (nmol/cm^2^)
(a)	for 0 s	10.34	801	70.6	5.85 ± 0.31	143
(b)	for 10 s	11.02	811	71.4	6.38 ± 0.47	145
(c)	for 20 s	11.27	797	72.0	6.47 ± 0.43	147
(d)	for 30 s	12.17	778	68.7	6.50 ± 0.51	148
(e)	for 40 s	10.90	739	65.8	5.30 ± 0.94	162

**Table 2:** Photovoltaic properties of DSSCs based on f-TNTAs with Au NPs and a scattering layer.

DSSCs Based on the f-TNTAs		*J_sc_* (mA/cm^2^)	*V_oc_* (mV)	*FF* (%)	*η* (%)	Dye Loading (nmol/cm^2^)
(a)	without both Au NPs and a scattering layer	10.34	801	70.6	5.85 ± 0.31	143
(b)	with Au NPs	12.17	778	68.7	6.50 ± 0.51	148
(c)	with a scattering layer	11.56	807	70.8	6.61 ± 0.37	153
(d)	with Au NPs and a scattering layer	13.10	780	69.7	7.12 ± 0.55	154

**Table 3:** Impedance properties of DSSCs based on f-TNTAs with Au NPs and a scattering layer.

DSSCs Based on the f-TNTAs		*R_s_* (Ω)	*R_1_* (Ω)	*R_2_* (Ω)
(a)	without Au NPs and a scattering layer	5.099	1.257	12.77
(b)	with Au NPs	5.039	1.088	11.68
(c)	with a scattering layer	5.059	1.098	11.75
(d)	with Au NPs and a scattering layer	4.942	1.071	10.98

## Data Availability

Data is contained within the article.

## Supplementary Materials

The following are available online at https://www.mdpi.com/2079-4991/11/2/328/s1, Figure S1. I-V curves of DSSCs based on the f-TNTAs DSSCs based on f-TNTAs with a TiO_2_ NPs layer or a scattering layer. Table S1. Photovoltaic properties of DSSCs based on f-TNTAs with a TiO_2_ NPs layer or a scattering layer.
