# Supplementary material for: Effect of Au Nanoparticles and Scattering Layer in Dye-Sensitized Solar Cells Based on Freestanding TiO2 Nanotube Arrays
Source: Nanomaterials (Basel). 2021 Jan 27;11(2):328. doi: 10.3390/nano11020328 (PMC7911132; doi:10.3390/nano11020328)
Supplement: Supplementary file 1 [file nanomaterials-11-00328-s001.pdf]

Supplementary Material

Effect of Au Nanoparticles and Scattering Layer in Dye-Sensitized Solar Cells Based on Freestanding TiO_2 Nanotube Arrays

Kang-Hun Lee [1,†], Seung-Hee Han [1,†], Ana Chuquer [2,†], Hwa-Young Yang [3,†], Jaehi Kim [4], Xuan-Hung Pham [4], Won-Ju Yun [5], Bong-Hyun Jun [4,*] and Won-Yeop Rho [1,*]

1 School of International Engineering and Science, Jeonbuk National University, 567, Baekje-daero, Deokjin-gu, Jeonju-si, Jeollabuk-do 54896, Korea; dlrkdgns343@naver.com (K.-H.L.); rinmin0616@naver.com (S.-H.H.)
2 School of Bioenvironmental Chemistry, Jeonbuk National University, 567, Baekje-daero, Deokjin-gu, Jeonju-si, Jeollabuk-do 54896, Korea; chuquerana@gmail.com (A.C.)
3 School of Energy and Chemical Engineering, Ulsan National Institute of Science and Technology (UNIST), 50 UNIST-gil, Eonyang-eup, Ulju-gun, Ulsan 44919, Korea; hyang@unist.ac.kr (H.-Y.Y.)
4 Department of Bioscience and Biotechnology, Konkuk University, 120, Neungdong-ro, Gwangjin-gu, Seoul 05029, Korea; susia45@gmail.com (J.K.); phamricky@gmail.com (X.-H.P.)
5 Department of Physics, Jeonbuk National University, 567, Baekje-daero, Deokjin-gu, Jeonju-si, Jeollabuk-do 54896, Korea; enneades@hanmail.net (W.-J.Y.)
* Correspondence: bjun@konkuk.ac.kr (B.-H.J.); rho7272@jbnu.ac.kr (W.-Y.R.)
† Kang-Hun Lee, Seung-Hee Han, Ana Chuquer, and Hwa-Young Yang contributed equally to this work.

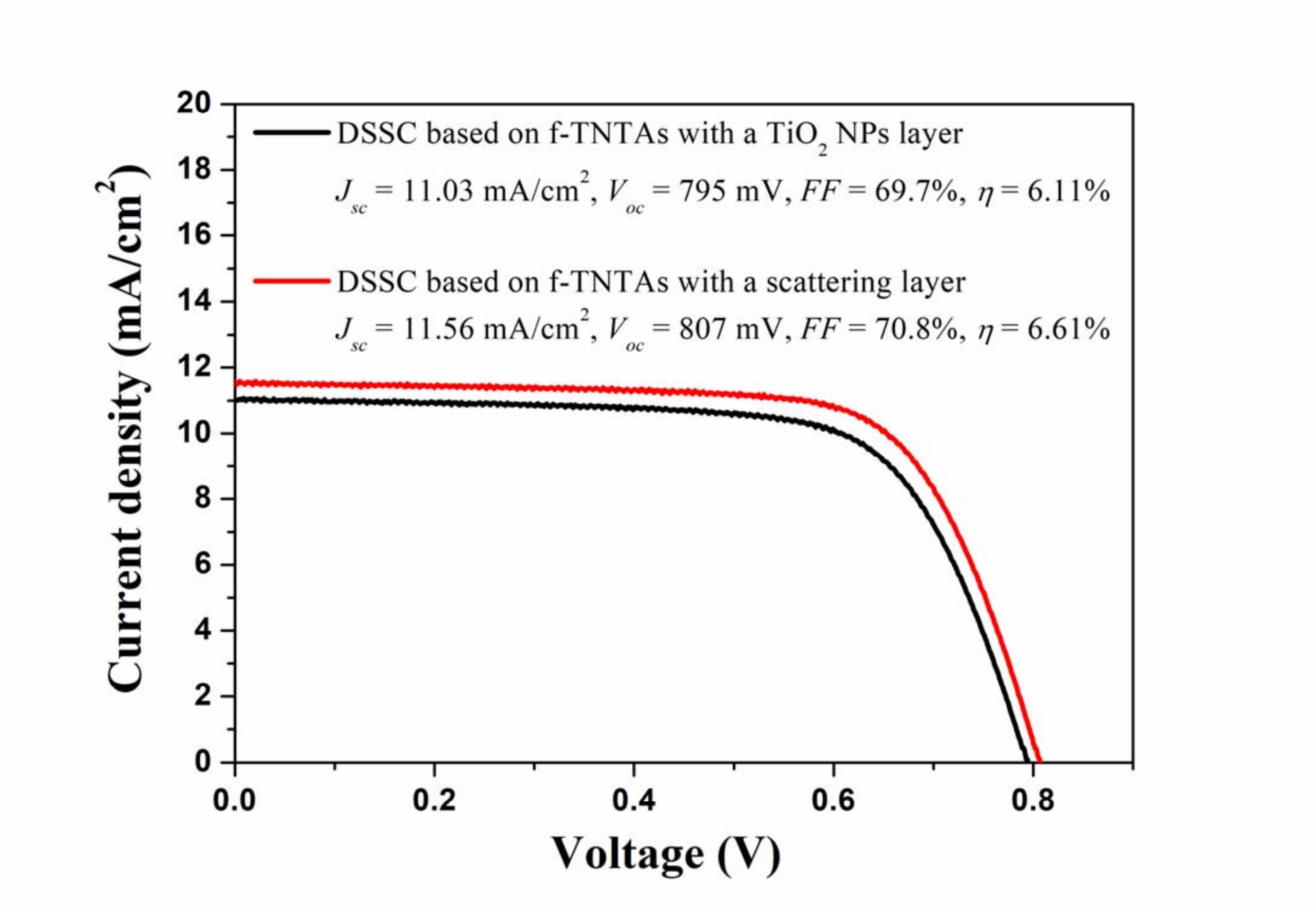

Figure S1. I-V curves of DSSCs based on the f-TNTAs DSSCs based on f-TNTAs with a TiO_2 NPs layer or a scattering layer.

Table S1. Photovoltaic properties of DSSCs based on f-TNTAs with a TiO_2 NPs layer or a scattering layer.

DSSCs based on the f-TNTAs		J_{sc} (mA/cm^2)	V_{oc} (mV)	FF (%)	η (%)	Dye Loading (nmol/cm^2)
(a)	with a TiO_2 NPs layer	11.03	795	69.7	6.11 ± 0.40	165
(b)	with a scattering layer	11.56	807	70.8	6.61 ± 0.37	153
